# Poly[di-μ-aqua-μ_4_-(pyrazine-2,5-dicarboxyl­ato)-dilithium(I)]

**DOI:** 10.1107/S1600536810050762

**Published:** 2010-12-08

**Authors:** Wojciech Starosta, Janusz Leciejewicz

**Affiliations:** a Institute of Nuclear Chemistry and Technology, ul.Dorodna 16, 03-195 Warszawa, Poland

## Abstract

In the title coordination polymer, [Li_2_(C_6_H_2_N_2_O_2_)(H_2_O)_2_]_*n*_ the pyrazine-2,5-dicarboxyl­ate dianionic ligand bridges two symmetry-independent Li^+^ ions using both its *N*,*O*-chelating sites. The carboxyl­ate O atom of one of them also bridges to another Li^+^ ion, while the second O atom of this group is bonded to another Li^+^ ion. Two symmetry-independent water O atoms participate also in the bridging system, which gives rise to a polymeric three-dimensional framework. Both Li^+^ ions show distorted trigonal–bipyramidal LiNO_4_ coordination geometries, with the N atom in an axial site in both cases. The packing is consolidated by O—H⋯O hydrogen bonds, which occur between water mol­ecules as donors and carboxyl­ate O atoms as acceptors.

## Related literature

For the crystal structures of transition metal complexes with the title ligand, see: Beobide *et al.* (2003 ▶); Xu *et al.* (2003 ▶); Beobide *et al.* (2006 ▶). For the structures of Cd and Zn complexes, see: Liu *et al.* (2009 ▶); Yang & Wu (2009 ▶); Yang *et al.* (2009 ▶). For the structures of polymeric lanthanide complexes, see: Zheng & Jin (2005 ▶); Yang *et al.* (2009 ▶). For the structure of a Th(IV) complex, see: Frisch & Cahill (2008 ▶). For the structure of an Sr(II) complex, see: Ptasiewicz-Bąk & Leciejewicz (1998*a* ▶). The structures of Li(I) complexes with pyrazine-2,3-dicarboxyl­ate and water ligands (Tombul *et al.*, 2008 ▶), 3-amino­pyrazine-2-carboxyl­ate and water ligands (Starosta & Leciejewicz, 2010*a* ▶) and pyrazine-2,3,5,6-tetra­carboxyl­ate and water ligands (Starosta & Leciejewicz, 2010*b* ▶) have been published. For the structure of pyrazine-2,5-dicarb­oxy­lic acid dihydrate, see: Ptasiewicz-Bąk & Leciejewicz (1998*b* ▶); Vishweshwar *et al.* (2002 ▶).
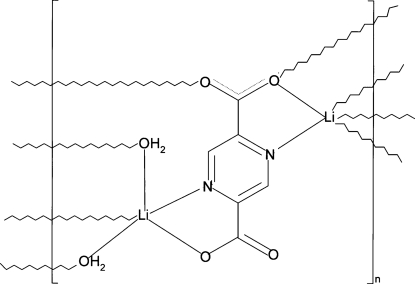


## Experimental

### 

#### Crystal data

[Li_2_(C_6_H_2_N_2_O_2_)(H_2_O)_2_]
*M* *_r_* = 216.01
Monoclinic, 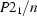

*a* = 7.2107 (14) Å
*b* = 7.3646 (15) Å
*c* = 15.327 (3) Å
β = 99.71 (3)°
*V* = 802.2 (3) Å^3^
*Z* = 4
Mo *K*α radiation
μ = 0.16 mm^−1^
*T* = 293 K
0.33 × 0.17 × 0.15 mm

#### Data collection

Kuma KM-4 four-circle diffractometer
Absorption correction: analytical (*CrysAlis RED*; Oxford Diffraction, 2008 ▶) *T*_min_ = 0.976, *T*_max_ = 0.985
2520 measured reflections
2348 independent reflections
1694 reflections with *I* > 2σ(*I*)
*R*_int_ = 0.069
3 standard reflections every 200 reflections  intensity decay: 0.6%

#### Refinement

*R*[*F* ^2^ > 2σ(*F* ^2^)] = 0.054
*wR*(*F* ^2^) = 0.161
*S* = 0.99
2348 reflections
161 parameters
H atoms treated by a mixture of independent and constrained refinement
Δρ_max_ = 0.73 e Å^−3^
Δρ_min_ = −0.62 e Å^−3^

### 

Data collection: *KM-4 Software* (Kuma, 1996 ▶); cell refinement: *KM-4 Software*; data reduction: *DATAPROC* (Kuma, 2001 ▶); program(s) used to solve structure: *SHELXS97* (Sheldrick, 2008 ▶); program(s) used to refine structure: *SHELXL97* (Sheldrick, 2008 ▶); molecular graphics: *SHELXTL* (Sheldrick, 2008 ▶); software used to prepare material for publication: *SHELXTL*.

## Supplementary Material

Crystal structure: contains datablocks I, global. DOI: 10.1107/S1600536810050762/hb5759sup1.cif

Structure factors: contains datablocks I. DOI: 10.1107/S1600536810050762/hb5759Isup2.hkl

Additional supplementary materials:  crystallographic information; 3D view; checkCIF report

## Figures and Tables

**Table 1:** Selected bond lengths (Å)

Li1—O1	1.958 (3)
Li1—O5	2.020 (3)
Li1—O6	2.077 (3)
Li1—O3^i^	2.131 (3)
Li1—N1	2.360 (3)
Li2—O6^ii^	1.981 (3)
Li2—O3	2.045 (3)
Li2—O5^iii^	2.056 (3)
Li2—O4^iv^	2.332 (4)
Li2—N2	2.129 (3)

Symmetry codes: (i) 
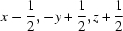
; (ii) 
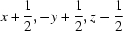
; (iii) 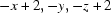; (iv) 
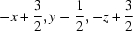
.

**Table 2:** Hydrogen-bond geometry (Å, °)

*D*—H⋯*A*	*D*—H	H⋯*A*	*D*⋯*A*	*D*—H⋯*A*
O6—H62⋯O2^v^	0.88 (3)	1.86 (3)	2.7210 (16)	165 (3)
O6—H61⋯O2^vi^	0.84 (4)	2.00 (4)	2.8351 (19)	170 (4)
O5—H52⋯O4^vii^	0.91 (3)	1.82 (3)	2.7292 (17)	175 (3)
O5—H51⋯O1^viii^	0.83 (3)	1.87 (3)	2.6842 (16)	169 (3)

Symmetry codes: (v) 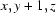; (vi) 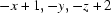; (vii) 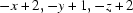; (viii) 
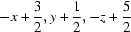
.

**Fig. 1.**
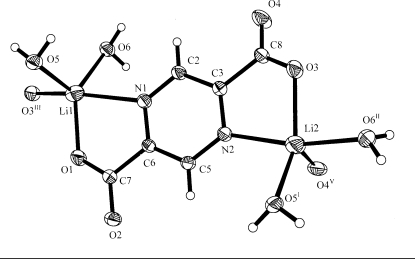
A structural unit of (I) with 50% probability displacement ellipsoids. Symmetry code: (i) -x + 2,-y,-z + 2. (ii) x + 1/2, -y + 1/2, z - 1/2. (iii) x - 1/2, -y + 1/2, z + 1/2. (iv) -x + 3/2, y + 1/2, -z + 3/2. (v) -x + 3/2, y - 1/2, -z + 3/2.

**Fig. 2.**
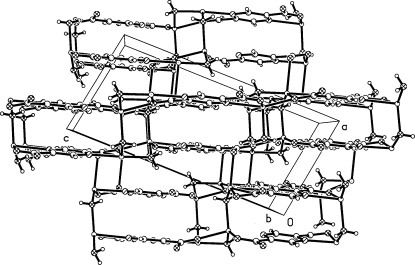
A fragment of a molecular layer.

## supplementary crystallographic information

### Comment

Metal complexes with pyrazine dicarboxylate ligands are of interest as precursors for new polymeric materials with a wide spectrum of potential applications. Owing to a pair of *N*,*O*-chelating sites localized at opposite terminals of the hetero-ring, pyrazine-2,5-dicarboxylate ligand shows a marked tendency to form coordination polymers. Structures with a variety of polymeric patterns have been reported in compounds with 3*d* transition metal ions (Xu *et al.*, 2003; Beobide *et al.* 2003; Beobide *et al.*, 2006); with a number of lanthanide ions (Zheng & Jin, 2005; with Cd(II) ion (Liu *et al.*, 2009; Yang & Wu, 2009); with Th(IV) ion (Frisch & Cahill, 2008) and with Sr(II) ion (Ptasiewicz-Bąk & Leciejewicz, 1998*b*). The asymmetric unit cell of the title complex, (I), contains a ligand dianion, two symmetry independent Li1 and Li2 ions and two symmetry independent O5 and O6 water molecules (Fig.1). The ligand molecule bridges the Li1 and Li2 ions using both its *N*,*O*-chelating sites; the carboxylate O2 atom remains coordination inactive. The O3 atom, which acts as bidentate, bridges the Li2 ion to the adjacent Li1^ii^ ion and with the coordinated water O6 atom gives rise to a molecular chain in which metal ions are bridged by the ligand on one side and two O atoms on the other. Water O5 atoms link the chains into molecular layers. (Fig. 2). The latter, bridged by carboxylate O4 atoms which link the ligands with Li2^iv^ ions in adjacent layers give rise to a three-dimensional framework. The coordination environment of the Li1 ion is composed of N1, O5, O3^iii^ atoms: they form together with the metal ion an equatorial plane (r.m.s. of 0.0021 (1) Å) of a distorted trigonal bipyramid; the O1 and O6 atoms are at its opposite apices. The Li2 ion together with coordinated O3, O4^v^ and O5^i^ atoms forms an equatorial plane (r.m.s. of 0.0307 (1) Å) of a distorted trigonal bipyramid, N2 and O6^ii^ atoms are at its apices. The observed Li—O bond distances fall in the range from 1.958 (3) to 2.131 (3)Å observed also in Li complexes with pyrazine carboxylate and water ligands (Tombul *et al.*, 2008; Starosta & Leciejewicz, 2010*a*, 2010*b*). The O4—Li2^iv^ bond distance is 2.332 (4) Å; the Li1—N1 and Li2—N2 bond lengths are 2.360 (3)Å and 2.129 (3) Å, respectively. The pyrazine ring is planar with r.m.s. of 0.0094 (1) Å, the carboxylate C7/O1/O2 and C8/O3/O4 groups make with it dihedral angles of 0.55 (20)° and 18.68 (17)°, respectively. Bond lengths and bond angles within the pyrazine ligand match those observed in the structure of the parent acid (Ptasiewicz-Bąk & Leciejewicz, 1998*a*; Vishweshwar *et al.*, 2002). Hydrogen bond network is composed of coordinated water molecules which are as donors and carboxylate O atoms which act as acceptors.

### Experimental

1 mmol of pyrazine-2,5-dicarboxylic acid dihydrate (Aldrich) dissolved in 30 ml of hot water and 2 mmols of lithium hydroxide (Aldrich) dissolved in 30 ml of hot water were mixed and boiled for 3 h under reflux with stirring. After cooling to room temperature, the solution was filtered and left to crystallize. After evaporation to dryness colourless blocks of (I) were found on the bottom of the reaction pot. They were washed with ethanol and dried in the air.

### Refinement

Water hydrogen atoms were located in a difference map and refined isotropically. H atoms attached to pyrazine-ring C atoms were positioned at calculated positions and treated as riding on the parent atoms, with C—H=0.93 Å and *U*_iso_(H)=1.2*U*_eq_(C).

### Crystal data

**Table:**

[Li_2_(C_6_H_2_N_2_O_2_)(H_2_O)_2_]	*F*(000) = 440
*M**_r_* = 216.01	*D*_x_ = 1.788 Mg m^−^^3^
Monoclinic, *P*2_1_/*n*	Mo *K*α radiation, λ = 0.71073 Å
Hall symbol: -P 2yn	Cell parameters from 25 reflections
*a* = 7.2107 (14) Å	θ = 6–15°
*b* = 7.3646 (15) Å	µ = 0.16 mm^−^^1^
*c* = 15.327 (3) Å	*T* = 293 K
β = 99.71 (3)°	Plates, colourless
*V* = 802.2 (3) Å^3^	0.33 × 0.17 × 0.15 mm
*Z* = 4	

### Data collection

**Table:**

Kuma KM-4 four-circle diffractometer	1694 reflections with *I* > 2σ(*I*)
Radiation source: fine-focus sealed tube	*R*_int_ = 0.069
graphite	θ_max_ = 30.1°, θ_min_ = 2.7°
profile data from ω/2θ scans	*h* = 0→10
Absorption correction: analytical (*CrysAlis RED*; Oxford Diffraction, 2008)	*k* = 0→10
*T*_min_ = 0.976, *T*_max_ = 0.985	*l* = −21→21
2520 measured reflections	3 standard reflections every 200 reflections
2348 independent reflections	intensity decay: 0.6%

### Refinement

**Table:**

Refinement on *F*^2^	Primary atom site location: structure-invariant direct methods
Least-squares matrix: full	Secondary atom site location: difference Fourier map
*R*[*F*^2^ > 2σ(*F*^2^)] = 0.054	Hydrogen site location: inferred from neighbouring sites
*wR*(*F*^2^) = 0.161	H atoms treated by a mixture of independent and constrained refinement
*S* = 0.99	*w* = 1/[σ^2^(*F*_o_^2^) + (0.1327*P*)^2^ + 0.0049*P*] where *P* = (*F*_o_^2^ + 2*F*_c_^2^)/3
2348 reflections	(Δ/σ)_max_ = 0.001
161 parameters	Δρ_max_ = 0.73 e Å^−^^3^
0 restraints	Δρ_min_ = −0.62 e Å^−^^3^

### Special details

**Table:**

Geometry. All e.s.d.'s (except the e.s.d. in the dihedral angle between two l.s. planes) are estimated using the full covariance matrix. The cell e.s.d.'s are taken into account individually in the estimation of e.s.d.'s in distances, angles and torsion angles; correlations between e.s.d.'s in cell parameters are only used when they are defined by crystal symmetry. An approximate (isotropic) treatment of cell e.s.d.'s is used for estimating e.s.d.'s involving l.s. planes.
Refinement. Refinement of *F*^2^ against ALL reflections. The weighted *R*-factor *wR* and goodness of fit *S* are based on *F*^2^, conventional *R*-factors *R* are based on *F*, with *F* set to zero for negative *F*^2^. The threshold expression of *F*^2^ > σ(*F*^2^) is used only for calculating *R*-factors(gt) *etc*. and is not relevant to the choice of reflections for refinement. *R*-factors based on *F*^2^ are statistically about twice as large as those based on *F*, and *R*- factors based on ALL data will be even larger.

### Fractional atomic coordinates and isotropic or equivalent isotropic displacement parameters (Å^2^)

**Table:**

	*x*	*y*	*z*	*U*_iso_*/*U*_eq_	
O5	0.92850 (16)	0.21032 (15)	1.23861 (7)	0.0235 (3)	
N2	0.85719 (16)	0.06063 (16)	0.86051 (8)	0.0178 (3)	
O3	0.99726 (17)	0.31016 (14)	0.76139 (7)	0.0248 (3)	
O6	0.54300 (17)	0.41912 (16)	1.12325 (8)	0.0248 (3)	
N1	0.76024 (18)	0.16459 (17)	1.02138 (8)	0.0194 (3)	
O1	0.65141 (17)	−0.08194 (16)	1.12869 (7)	0.0258 (3)	
C7	0.69244 (18)	−0.14435 (18)	1.05880 (8)	0.0171 (3)	
C3	0.85787 (18)	0.23438 (19)	0.88494 (9)	0.0167 (3)	
C8	0.9150 (2)	0.37044 (19)	0.82054 (9)	0.0194 (3)	
O2	0.68766 (17)	−0.30725 (14)	1.03608 (7)	0.0252 (3)	
O4	0.8671 (2)	0.53125 (16)	0.83062 (9)	0.0342 (3)	
C5	0.80318 (19)	−0.06117 (19)	0.91591 (9)	0.0177 (3)	
H5	0.7990	−0.1835	0.9006	0.021*	
C6	0.75335 (17)	−0.00880 (18)	0.99566 (8)	0.0155 (3)	
C2	0.8103 (2)	0.28635 (19)	0.96561 (9)	0.0202 (3)	
H2	0.8136	0.4087	0.9808	0.024*	
Li2	0.9486 (5)	0.0403 (4)	0.7358 (2)	0.0360 (7)	
Li1	0.6732 (4)	0.1742 (4)	1.1630 (2)	0.0298 (6)	
H51	0.908 (4)	0.263 (4)	1.284 (2)	0.063 (9)*	
H62	0.609 (4)	0.503 (4)	1.1012 (17)	0.049 (7)*	
H52	0.994 (4)	0.294 (4)	1.2126 (18)	0.051 (7)*	
H61	0.472 (5)	0.375 (5)	1.079 (3)	0.093 (12)*	

### Atomic displacement parameters (Å^2^)

**Table:**

	*U*^11^	*U*^22^	*U*^33^	*U*^12^	*U*^13^	*U*^23^
O5	0.0301 (6)	0.0199 (5)	0.0225 (5)	−0.0017 (4)	0.0104 (4)	−0.0037 (4)
N2	0.0191 (5)	0.0162 (5)	0.0190 (5)	0.0008 (4)	0.0059 (4)	0.0006 (4)
O3	0.0301 (6)	0.0227 (5)	0.0246 (5)	0.0010 (4)	0.0137 (4)	0.0013 (4)
O6	0.0298 (6)	0.0216 (5)	0.0246 (5)	−0.0021 (4)	0.0096 (4)	0.0010 (4)
N1	0.0229 (6)	0.0172 (6)	0.0191 (5)	0.0018 (4)	0.0064 (4)	−0.0001 (4)
O1	0.0365 (6)	0.0212 (5)	0.0230 (5)	0.0003 (4)	0.0148 (5)	0.0015 (4)
C7	0.0148 (6)	0.0174 (6)	0.0191 (6)	−0.0001 (5)	0.0033 (5)	0.0010 (5)
C3	0.0156 (6)	0.0166 (6)	0.0182 (6)	0.0019 (4)	0.0037 (5)	0.0015 (4)
C8	0.0213 (7)	0.0169 (6)	0.0211 (6)	0.0007 (5)	0.0063 (5)	0.0025 (5)
O2	0.0335 (6)	0.0178 (5)	0.0259 (5)	−0.0039 (4)	0.0094 (4)	−0.0006 (4)
O4	0.0516 (8)	0.0171 (5)	0.0400 (7)	0.0061 (5)	0.0249 (6)	0.0042 (5)
C5	0.0197 (6)	0.0152 (6)	0.0192 (6)	−0.0008 (5)	0.0057 (5)	−0.0004 (4)
C6	0.0145 (6)	0.0145 (6)	0.0178 (6)	0.0008 (4)	0.0035 (5)	0.0014 (4)
C2	0.0264 (7)	0.0145 (6)	0.0211 (6)	0.0025 (5)	0.0082 (5)	0.0003 (5)
Li2	0.053 (2)	0.0239 (14)	0.0359 (16)	−0.0019 (13)	0.0218 (15)	−0.0027 (11)
Li1	0.0305 (14)	0.0250 (13)	0.0332 (14)	0.0005 (11)	0.0033 (11)	−0.0046 (11)

### Geometric parameters (Å, °)

**Table:**

O5—Li2^i^	2.056 (4)	O6—H62	0.88 (3)
O3—Li1^ii^	2.131 (3)	O6—H61	0.84 (4)
O6—Li2^iii^	1.981 (3)	N1—C2	1.3303 (18)
O4—Li2^iv^	2.332 (4)	N1—C6	1.3348 (18)
Li1—O1	1.958 (3)	O1—C7	1.2463 (17)
Li1—O5	2.020 (3)	C7—O2	1.2481 (17)
Li1—O6	2.077 (3)	C7—C6	1.5064 (18)
Li1—O3^iii^	2.131 (3)	C3—C2	1.3913 (18)
Li1—N1	2.360 (3)	C3—C8	1.5117 (19)
Li2—O6^ii^	1.981 (3)	C8—O4	1.2506 (19)
Li2—O3	2.045 (3)	C5—C6	1.3857 (18)
Li2—O5^i^	2.056 (3)	C5—H5	0.9300
Li2—O4^v^	2.332 (4)	C2—H2	0.9300
Li2—N2	2.129 (3)	Li2—Li1^ii^	2.983 (4)
O5—H51	0.83 (3)	Li2—Li1^i^	3.303 (5)
O5—H52	0.91 (3)	Li1—Li2^iii^	2.983 (4)
N2—C3	1.3330 (18)	Li1—Li2^i^	3.303 (5)
N2—C5	1.3376 (17)	Li1—H61	2.30 (4)
O3—C8	1.2458 (17)		
			
Li1—O5—Li2^i^	108.24 (14)	O3—Li2—N2	80.13 (12)
Li1—O5—H51	105 (2)	O5^i^—Li2—N2	94.63 (14)
Li2^i^—O5—H51	113 (2)	O6^ii^—Li2—O4^v^	94.45 (15)
Li1—O5—H52	109.1 (17)	O3—Li2—O4^v^	103.58 (15)
Li2^i^—O5—H52	117.0 (18)	O5^i^—Li2—O4^v^	114.53 (15)
H51—O5—H52	103 (3)	N2—Li2—O4^v^	88.10 (13)
C3—N2—C5	116.94 (12)	O6^ii^—Li2—Li1^ii^	43.94 (9)
C3—N2—Li2	109.43 (13)	O3—Li2—Li1^ii^	45.59 (9)
C5—N2—Li2	133.63 (13)	O5^i^—Li2—Li1^ii^	117.39 (15)
C8—O3—Li2	113.48 (13)	N2—Li2—Li1^ii^	123.78 (15)
C8—O3—Li1^ii^	155.36 (13)	O4^v^—Li2—Li1^ii^	114.10 (14)
Li2—O3—Li1^ii^	91.16 (13)	O6^ii^—Li2—Li1^i^	95.92 (14)
Li2^iii^—O6—Li1	94.61 (13)	O3—Li2—Li1^i^	105.87 (15)
Li2^iii^—O6—H62	121.1 (18)	O5^i^—Li2—Li1^i^	35.51 (9)
Li1—O6—H62	118.3 (19)	N2—Li2—Li1^i^	88.18 (13)
Li2^iii^—O6—H61	120 (3)	O4^v^—Li2—Li1^i^	149.19 (14)
Li1—O6—H61	95 (3)	Li1^ii^—Li2—Li1^i^	93.17 (11)
H62—O6—H61	105 (3)	O1—Li1—O5	107.76 (15)
C2—N1—C6	117.11 (12)	O1—Li1—O6	138.27 (17)
C2—N1—Li1	135.56 (12)	O5—Li1—O6	112.16 (15)
C6—N1—Li1	107.34 (11)	O1—Li1—O3^iii^	102.21 (15)
C7—O1—Li1	124.49 (14)	O5—Li1—O3^iii^	100.41 (14)
O1—C7—O2	126.51 (13)	O6—Li1—O3^iii^	82.36 (12)
O1—C7—C6	116.42 (12)	O1—Li1—N1	75.27 (11)
O2—C7—C6	117.07 (12)	O5—Li1—N1	100.00 (14)
N2—C3—C2	121.60 (12)	O6—Li1—N1	86.19 (12)
N2—C3—C8	116.19 (11)	O3^iii^—Li1—N1	159.18 (16)
C2—C3—C8	122.21 (12)	O1—Li1—Li2^iii^	139.07 (16)
O3—C8—O4	127.07 (13)	O5—Li1—Li2^iii^	101.09 (13)
O3—C8—C3	117.04 (13)	O6—Li1—Li2^iii^	41.45 (9)
O4—C8—C3	115.82 (12)	O3^iii^—Li1—Li2^iii^	43.25 (9)
C8—O4—Li2^iv^	104.05 (13)	N1—Li1—Li2^iii^	127.64 (14)
N2—C5—C6	121.33 (13)	O1—Li1—Li2^i^	71.81 (11)
N2—C5—H5	119.3	O5—Li1—Li2^i^	36.25 (8)
C6—C5—H5	119.3	O6—Li1—Li2^i^	148.18 (15)
N1—C6—C5	121.63 (12)	O3^iii^—Li1—Li2^i^	103.68 (13)
N1—C6—C7	116.40 (11)	N1—Li1—Li2^i^	95.21 (12)
C5—C6—C7	121.96 (12)	Li2^iii^—Li1—Li2^i^	128.23 (11)
N1—C2—C3	121.33 (13)	O1—Li1—H61	117.0 (10)
N1—C2—H2	119.3	O5—Li1—H61	131.5 (10)
C3—C2—H2	119.3	O6—Li1—H61	21.3 (10)
O6^ii^—Li2—O3	86.97 (13)	O3^iii^—Li1—H61	88.0 (9)
O6^ii^—Li2—O5^i^	95.80 (14)	N1—Li1—H61	75.4 (9)
O3—Li2—O5^i^	141.4 (2)	Li2^iii^—Li1—H61	54.9 (10)
O6^ii^—Li2—N2	167.10 (18)	Li2^i^—Li1—H61	164.0 (10)

Symmetry codes: (i) −*x*+2, −*y*, −*z*+2; (ii) *x*+1/2, −*y*+1/2, *z*−1/2; (iii) *x*−1/2, −*y*+1/2, *z*+1/2; (iv) −*x*+3/2, *y*+1/2, −*z*+3/2; (v) −*x*+3/2, *y*−1/2, −*z*+3/2.

### Hydrogen-bond geometry (Å, °)

**Table:**

*D*—H···*A*	*D*—H	H···*A*	*D*···*A*	*D*—H···*A*
O6—H62···O2^vi^	0.88 (3)	1.86 (3)	2.7210 (16)	165 (3)
O6—H61···O2^vii^	0.84 (4)	2.00 (4)	2.8351 (19)	170 (4)
O5—H52···O4^viii^	0.91 (3)	1.82 (3)	2.7292 (17)	175 (3)
O5—H51···O1^ix^	0.83 (3)	1.87 (3)	2.6842 (16)	169 (3)

Symmetry codes: (vi) *x*, *y*+1, *z*; (vii) −*x*+1, −*y*, −*z*+2; (viii) −*x*+2, −*y*+1, −*z*+2; (ix) −*x*+3/2, *y*+1/2, −*z*+5/2.

## References

[bb1] Beobide, G., Castillo, O., Luque, A., Garcia-Couceiro, U., Garcia-Teran, J. P. & Roman, P. (2006). *Inorg. Chem.* **45**, 5367–5382. 10.1021/ic060221r 16813400

[bb2] Beobide, G., Castillo, O., Luque, A., Garcia-Couceiro, U., Garcia-Teran, J. P., Roman, P. & Lezama, L. (2003). *Inorg. Chem. Commun.* **6**, 1224–1227.

[bb3] Frisch, M. & Cahill, C. L. (2008). *Cryst. Growth Des.* **8**, 2921–2926.

[bb4] Kuma (1996). *KM-4 Software* Kuma Diffraction Ltd, Wrocław, Poland.

[bb5] Kuma (2001). *DATAPROC* Kuma Diffraction Ltd, Wrocław, Poland.

[bb6] Liu, F.-Q., Li, R.-X., Deng, Y.-Y., Li, W.-H., Ding, N.-X. & Liu, G.-Y. (2009). *J. Organomet. Chem.* **694**, 3653–3659.

[bb7] Oxford Diffraction (2008). *CrysAlis RED* Oxford Diffraction Ltd, Yarnton, England.

[bb8] Ptasiewicz-Bąk, H. & Leciejewicz, J. (1998*a*). *J. Coord. Chem.* **44**, 299–309.

[bb9] Ptasiewicz-Bąk, H. & Leciejewicz, J. (1998*b*). *J. Coord. Chem.* **44**, 237–246.

[bb10] Sheldrick, G. M. (2008). *Acta Cryst.* A**64**, 112–122. 10.1107/S0108767307043930 18156677

[bb11] Starosta, W. & Leciejewicz, J. (2010*a*). *Acta Cryst.* E**66**, m744–m745. 10.1107/S1600536810020647 PMC3006785 21587683

[bb12] Starosta, W. & Leciejewicz, J. (2010*b*). *Acta Cryst.* E**66**, m1561–m1562. 10.1107/S1600536810045903 PMC3011810 21589251

[bb13] Tombul, M., Güven, K. & Büyükgüngör, O. (2008). *Acta Cryst.* E**64**, m491–m492. 10.1107/S1600536808004467 PMC2960851 21201874

[bb14] Vishweshwar, P., Nangia, A. & Lynch, V. M. (2002). *J. Org. Chem.* **67**, 556–565. 10.1021/jo0162484 11798330

[bb15] Xu, H. T., Zheng, N. W., Yang, R. Y., Li, Z. Q. & Jin, X. L. (2003). *Inorg. Chim. Acta*, **349**, 265–268.

[bb16] Yang, P. & Wu, J.-Z. (2009). *Acta Cryst.* C**65**, m4–m6. 10.1107/S0108270108040080 19129597

[bb17] Yang, P., Wu, J.-Z. & Yu, V. (2009). *Inorg. Chim. Acta*, **362**, 1907–1912.

[bb19] Zheng, X-J. & Lin, J-P. (2005). *J. Chem. Crystallogr.* **35**, 865–869.

